# Volumineux hématome spontané sous capsulaire hépatique rompu: à propos d’un cas à Bamako

**DOI:** 10.11604/pamj.2019.34.187.20096

**Published:** 2019-12-09

**Authors:** Mahamadou Malle, Youssouf Kone

**Affiliations:** 1 Service de Radiologie, Hôpital Régional de Gao, Gao, Mali; 2 Service de Radiologie, Centre Hospitalier Universitaire du Point « G » de Bamako, Bamako, Mali

**Keywords:** Hématome sous-scapulaire, hépatique-spontané, rompu, scanner, Subcapsular hematoma of the liver, spontaneous, ruptured, scanner

## Abstract

Rupture of spontaneous subcapsular hematoma of the liver is very rare and therefore it has been rarely described in the literature. It generally occurs among women with underlying liver disease or often, during pregnancy, in women with HELLP syndrome. We here report the case of a 60-year old man with no particular past medical history presenting with large subcapsular haematoma of the liver. He was admitted to the Emergency Department with moderate-intensity right hypochondre pain evolving over the last week, which was aggravated, two days before, by influenza-like illness associating cough, yellowish sputum and sweatings. Clinical examination showed abdominal tenderness on palpation of the right hypochondre and hypogastrium. The remainder of the clinic examination was normal. Laboratory tests showed hemoglobin level 7.8g/dl, MCV: 79.9, reticulocyte count 48.9. computed tomography (CT) scan showed voluminous subcapsular hematoma of the liver measuring 17cm in height, 14cm in cross-sectional diameter and 5cm in thickness and a hepatic nodule measuring 10mm (A) with hemoperitoine of great abundance without active bleeding (B). The patient underwent visceral surgery (laparotomy) with favorable postoperative outcome.

## Image en médecine

L'hématome sous-capsulaire spontané rompu du foie est un phénomène très rare et très peu décrit dans la littérature. Ils surviennent généralement chez les femmes au cours d'une pathologie hépatique sous-jacente ou au décours d'une grossesse souvent dans le cadre d'un HELLP (Hemolysis, Elevated Liver enzymes, Low Platelet count) syndrome. Nous rapportons le cas d'un volumineux hématome sous capsulaire hépatique chez un homme de 60 ans sans antécédents pathologiques connus, admis aux urgences pour douleur de l'hypochondre droit évoluant depuis une semaine d'intensité modérée qui s'est aggravé il y a deux jours par un syndrome grippal associant toux, une expectoration jaunâtre des sueurs. A l'examen clinique nous avons retrouvé un abdomen sensible à la palpation de l'hypochondre droit et de l'hypogastre, le reste de l'examen clinique était normal. A la biologie le taux d'hémoglobine était à 7,8g/dl, VGM: 79,9, les reticulocytes à 48,9. Le scanner a montré un volumineux hématome sous capsulaire hépatique de 17cm de hauteur, 14cm de diamètre transversale et 5cm d'épaisseur et un nodule hépatique de 10mm (A) avec un hémopéritoine de grande abondance sans saignement actif (B). Le patient a bénéficié d'une laparotomie en chirurgie viscérale avec des suites post opératoires favorables.

**Figure 1:**
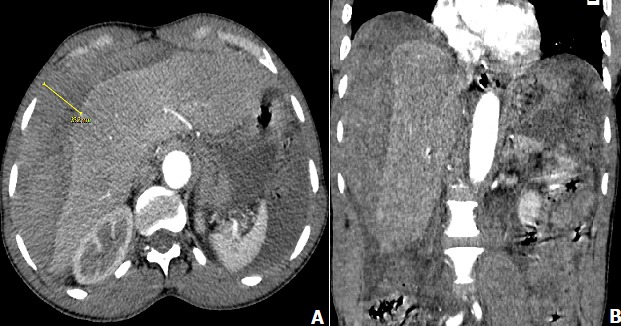
A) coupe axiale montrant un volumineux hématome sous capsulaire hépatique (flèche); B) coupe coronale montrant hémoperitoine de grande abondance sans saignement actif

